# Complete Genome Sequences of Newcastle Disease Virus Isolates from Backyard Chickens in Northern India

**DOI:** 10.1128/MRA.00467-19

**Published:** 2019-07-03

**Authors:** Sushila Maan, Sunil K. Mor, Naresh Jindal, Vinay G. Joshi, Chintu Ravishankar, Vikash K. Singh, Rajasekhar Ravindran, Niranjana Sahoo, Jessica Radzio-Basu, Megan A. Schilling, Walter R. McVey, Nand K. Mahajan, Vivek Kapur, Sagar M. Goyal

**Affiliations:** a Department of Animal Biotechnology, College of Veterinary Sciences, Lala Lajpat Rai University of Veterinary and Animal Science, Hisar, India; b Department of Veterinary Public Health & Epidemiology, College of Veterinary Sciences, Lala Lajpat Rai University of Veterinary and Animal Science, Hisar, India; c Department of Veterinary Population Medicine, College of Veterinary Medicine, University of Minnesota, St. Paul, Minnesota, USA; d Department of Veterinary Microbiology, College of Veterinary and Animal Sciences, Kerala Veterinary and Animal Sciences University, Pookode, Kerala, India; e College of Veterinary Science and Animal Husbandry, Orissa University of Agriculture & Technology, Bhubaneswar, Odisha, India; f The Huck Institute of the Life Sciences, Department of Epidemiology and Preventive Medicine, The Pennsylvania State University, University Park, Pennsylvania, USA; g The Applied Biological and Biosecurity Research Laboratory, The Pennsylvania State University, University Park, Pennsylvania, USA; h Department of Animal Science, The Pennsylvania State University, University Park, Pennsylvania, USA; DOE Joint Genome Institute

## Abstract

The molecular characterization of three Newcastle disease viruses (NDV) isolated from backyard chickens in the state of Haryana, India, was undertaken. Two genotype II strains and one genotype XIIIc class II isolate with genome sizes of 15,186 and 15,192 nucleotides (nt), respectively, were identified.

## ANNOUNCEMENT

Emerging and reemerging respiratory diseases in poultry, such as velogenic viscerotropic Newcastle disease (vvND), present a major threat to animal health worldwide, especially in countries with large backyard poultry sectors, including India. The disease is caused by Newcastle disease virus (NDV), a nonsegmented negative-sense single-stranded RNA virus of the species *Avian avulavirus 1* (AAvV-1) of the genus *Orthoavulavirus* of the *Paramyxoviridae* family (1). AAvV-1 has been reported to have a genome size of either 15,186 nucleotides (nt), 15,192 nt, or 15,198 nt, in each case encoding six structural proteins, nucleocapsid protein (NP), phosphoprotein (P), matrix protein (M), fusion protein (F), hemagglutinin-neuraminidase (HN), and large polymerase (L), arranged from 3′ to 5′ (1, 2). Based on genetic analysis, AAvV-1 strains are classified into two classes (I and II), with viruses representing class I being generally nonpathogenic for chickens and members of class II causing disease (2, 3). Class II is divided into 18 different genotypes (I to XVIII), some of which are further divided into subgenotypes, e.g., a, b, c, etc. (1–3).

The viruses characterized in this investigation were isolated from brain tissues of two nonvaccinated backyard chickens collected in 2016 at the Lala Lajpat Rai University of Veterinary and Animal Science in Hisar, India. Viral RNA from these isolates was extracted using TRIzol (Invitrogen) followed by an RNeasy minikit (Qiagen). The purified viral RNA was then quantified with a Qubit 2 fluorometer (Invitrogen), and the cDNA was synthesized using the SuperScript III first-strand synthesis system (Invitrogen) followed by Nextera XT library preparation (Illumina). The DNA concentrations and size distributions of the prepared libraries were checked on a fragment analyzer system (FSv2-CE2F; Advanced Analytical Technologies) using a high-sensitivity next-generation sequencing (NGS) fragment analysis kit. MiSeq 300-bp paired-end sequencing (Illumina) of qualified libraries was performed. Following sequencing, primer adaptor contamination was removed using Trimmomatic 0.38, and reads with a Phred quality score of >20 were selected for assembly. A total of 524,931 and 2,781,765 reads for samples 1 and 2, respectively, were assembled using CLC Genomics Workbench 11.0.1 with default parameters. Interestingly, the analysis of sample 1 identified coinfection with two NDV genomes of 15,186 nt (28,299 reads, 281-fold coverage) and 15,192 nt (295,344 reads, 2,931-fold coverage), here named NDV/DesiFowl/IND/HR212A and NDV/DesiFowl/IND/HR212B, respectively. A complete NDV genome (NDV/DesiFowl/IND/HR213) of 15,186 nt was assembled from 1,813,879 reads with an average depth of coverage of 18,786 from the second sample. The genomes were annotated using Geneious 11.1.5 and the ORFfinder algorithm (https://www.ncbi.nlm.nih.gov/orffinder/), and the untranscribed regions (UTRs) were confirmed based on alignment with previously published NDV sequences (GenBank accession numbers KC844235, KY774445, and KX345397) with coverage at the 3′ and 5′ UTRs greater than 10× for NDV/DesiFowl/IND/HR212B and NDV/DesiFowl/IND/HR213 and 4× for NDV/DesiFowl/IND/HR212A.

The analysis revealed that the NDV/DesiFowl/IND/HR212A and NDV/DesiFowl/IND/HR213 sequences had 99.12 to 99.86% nucleotide identities with genotype II vaccine strains, such as the LaSota strain (GenBank accession number KC844235), based on both F gene and complete genome alignments. In contrast, the complete genome of NDV/DesiFowl/IND/HR212B showed 95.14 to 97.39% nucleotide identities with genotype XIII sequences reported from India and 97.17 to 97.35% nucleotide identities with subgenotype XIIIc sequences reported recently from eastern states in India, e.g., the NDV/Chicken/Pandu/2015 (GenBank accession number KY774445) (4) and NDV/Chicken/Kamrup/07/14 (GenBank accession number KX345397) strains (5). The analysis suggested that strain NDV/DesiFowl/IND/HR212B represents a virulent pathotype, containing the ^112^RRQKRF^117^ sequence at the C terminus of the F protein cleavage site, which is known to be a major determinant of NDV pathogenicity (6, 7), while NDV/DesiFowl/IND/HR212A and NDV/DesiFowl/IND/HR213 both contain the ^112^GRQGRL^117^ sequence, suggesting that they represent lentogenic strains of NDV (8). Altogether, the results show that a single sample contained both vaccine-type (genotype II) and wild-type (genotype XIIIc) NDV, highlighting a need for unbiased characterization of potential mixed infections by next-generation sequencing approaches. Importantly, the results also suggest that genotype XIIIc NDV strains are present and are associated with diseased backyard chickens in India.

### Data availability.

The complete genome sequences of all three isolates have been deposited in GenBank with the accession numbers MK796808 (NDV/DesiFowl/IND/HR212A), MK796809 (NDV/DesiFowl/IND/HR212B), and MK796810 (NDV/DesiFowl/IND/HR213), and the raw FASTQ files have been deposited in the Sequence Read Archive with BioProject number PRJNA544371. The SRA accession numbers of the raw FASTQ files of samples 1 and 2 are SRX5886905 and SRX5886904, respectively.
